# Supplementary material for: LncRNA ZNF582-AS1 Expression and Methylation in Breast Cancer and Its Biological and Clinical Implications
Source: Cancers (Basel). 2022 Jun 4;14(11):2788. doi: 10.3390/cancers14112788 (PMC9179509; doi:10.3390/cancers14112788)
Supplement: Supplementary file 1 [file cancers-14-02788-s001.zip › cancers-1712720-supplementary.pdf]

Supplementary Figure S1.

A. Associations between overall survival and each CpG site methylation (high versus low) in the ZNF582-AS1 promoter by Cox regression

Probe	Hazards ratio	P value
cg01772700	0.767	0.438
cg24733179	0.652	0.205
cg11740878	1.159	0.660
cg09568464	1.190	0.602
cg02763101	1.541	0.195
cg22647407	1.182	0.614
cg08464824	1.567	0.181
cg13916740	1.011	0.973
cg24039631	1.223	0.550
cg20984085	1.354	0.362
cg25267765	1.729	0.120
cg07135042	1.693	0.127
cg07778983	0.982	0.956

B. Associations between disease-free survival and each CpG site methylation (high versus low) in the ZNF582-AS1 promoter by Cox regression

Probe	Hazards ratio	P value
cg01772700	0.791	0.424
cg24733179	0.893	0.699
cg11740878	0.737	0.305
cg09568464	1.046	0.879
cg02763101	1.321	0.342
cg22647407	1.150	0.634
cg08464824	1.513	0.161
cg13916740	1.080	0.794
cg24039631	0.905	0.736
cg20984085	1.463	0.197
cg25267765	1.404	0.259
cg07135042	1.202	0.530
cg07778983	0.993	0.980

C. Associations of overall and disease-free survival with average methylation of all the CpG sites (high versus low) in the ZNF582-AS1 promoter by Cox regression

	Hazards ratio	P value
OS	1.6641	0.1405418
DFS	1.0473	0.874645

D. Kaplan-Meier overall survival curves by levels of ZNF582-AS1 promoter methylation (average high, mid, and low)

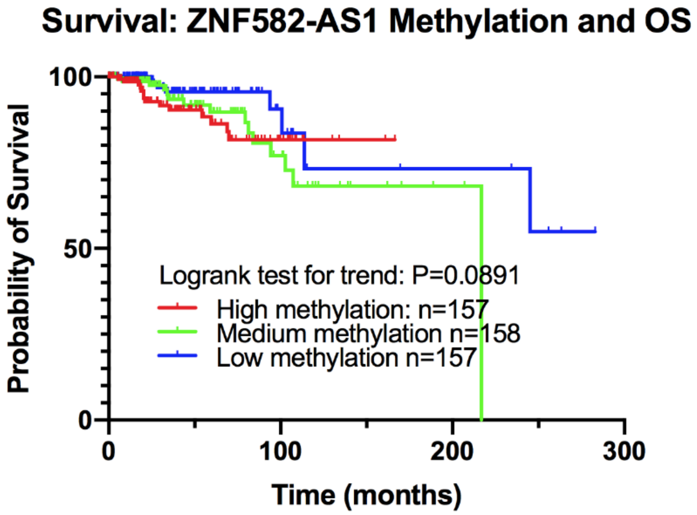

E. Kaplan-Meier disease-free survival curves by levels of ZNF582-AS1 promoter methylation (average high, mid, and low)

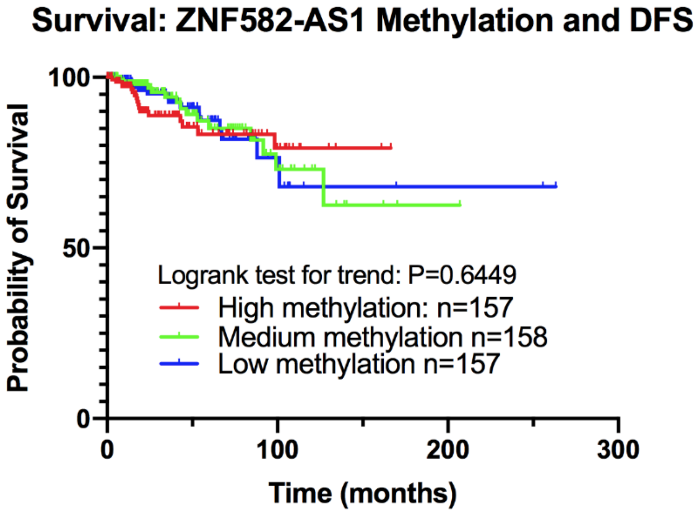

Supplementary Figure S2. Predicted binding between has-miR-940 and PTEN mRNA

http://www.targetscan.org/

	Predicted consequential pairing of target region (top) and miRNA (bottom)
Position 5974-5980 of PTEN 3' UTR<br><br>hsa-miR-940	5' ...CACCUUUAGGAUUUUCUGCCUAC...<br>|||||| 3' CCCCUCGCCCCCGGGACGGAA

Supplementary Table S1. Information on GEO datasets

Accession ID	Chip model	Platforms	No. of Patients	With OS data	With DFS data
GSE1456	Affymetrix Human Genome U133 A and B	GPL96 and GPL97	159	159	159
GSE16446	Affymetrix U133 plus 2.0	GPL570	120	107	107
GSE19615	Affymetrix U133 plus 2.0	GPL570	115	0	115
GSE20685	Affymetrix U133 plus 2.0	GPL570	327	327	307
GSE21653	Affymetrix U133 plus 2.0	GPL570	266	0	252
GSE31448	Affymetrix U133 plus 2.0	GPL570	353	0	250
GSE42568	Affymetrix U133 plus 2.0	GPL570	104	104	104
GSE4922	Affymetrix Human Genome U133 A and B	GPL96 and GPL97	289	0	249
GSE88770	Affymetrix U133 plus 2.0	GPL570	117	117	0
Total			1,850	814	1,543

Supplementary Table S2. Genes in the *ZNF582-AS1* expression signature

Gene symbol	Fold change	Adjusted p value
CYP2A6	7.489748685	0.01569974
GRIA1	4.90043538	0.010127825
SLC14A2	4.630868079	0.001345177
NCRNA00110	4.591525468	2.44877E-06
SLC4A4	4.00503011	0.001946918
DIRAS3	3.884333116	0.016662352
C8orf34	3.577023615	0.020399521
CYP4Z1	3.480705564	1.58891E-06
SMYD1	3.34790465	0.003080398
PIP	3.290811344	0.032983363
GCM1	3.290140824	0.028260244
FLJ37543	3.228508783	0.039493427
DDX25	3.146977982	0.014296828
CLDN19	3.122542923	1.37968E-08
GRIA2	2.882744063	0.000254737
COL17A1	2.845192173	1.88275E-06
CMA1	2.824538389	1.59941E-09
COX6B2	2.790800155	0.009040269
CD207	2.76983469	0.000551065
NPHS1	2.748531094	0.026031326
NTF3	2.702277594	0.003708599
TAC1	2.683219415	0.032415048
FGF14	2.625928047	0.006018955
STAB2	2.619950256	0.004092058
SCN3A	2.56949998	0.013675254
CST4	2.532061708	0.025436017
C7	2.532057704	2.08911E-09
KIRREL3	2.505063536	0.028396331
FXYD1	2.499628678	6.5228E-13
CD300LG	2.487359017	1.14127E-07
CNR1	2.484263172	0.002695623
SLC7A3	2.468877676	1.1425E-05
NOG	2.43194802	0.032092326
MMP27	2.425174428	0.005692847
PTGDR	2.420325698	0.001969993
MYH11	2.419907253	0.00010118
FIGF	2.410758768	1.59101E-05

TMC3	2.40831973	0.028416102
ZNF385D	2.406902308	1.50066E-05
SEMA6D	2.397240195	0.000388638
CTSG	2.385204312	4.75641E-09
PLAC9P1	2.383016569	1.14882E-05
GFAP	2.382410988	5.75561E-05
SLC18A2	2.377495374	0.00321158
FOSB	2.370004152	9.64797E-07
NPY5R	2.366376286	0.005931518
VIPR2	2.365223438	0.00385179
SCARA5	2.358879282	1.85023E-06
FCER1A	2.348894806	1.02454E-12
PCAT18	2.315122907	2.08525E-08
PAK7	2.307388239	8.51001E-08
ZNF385B	2.304842865	2.56893E-06
SLC16A12	2.299123189	0.000862141
ABCA8	2.295845087	8.33476E-11
CCDC108	2.295018996	2.49299E-05
NDP	2.279616676	0.001780493
ABCA10	2.269606101	2.37392E-10
SCUBE2	2.264033879	8.14516E-10
IGFN1	2.262824443	0.001195826
SCN3B	2.259963451	1.03022E-05
OGN	2.253203302	0.000750242
C14orf180	2.249363925	5.0777E-05
GLYAT	2.24598081	0.000146759
TMEM132C	2.241472767	6.05356E-08
RERGL	2.23310385	0.001396149
CASP12	2.227823271	7.05722E-10
BLK	2.225792769	0.044018246
C5orf38	2.225508384	2.5073E-06
ZNF582	2.224584785	1.26331E-87
CD1A	2.216180509	0.009320869
NTRK2	2.204780562	0.000180034
SDPR	2.196074348	4.3755E-12
ADH1A	2.189986323	0.000110082
AK5	2.187157439	4.62641E-07
HEPN1	2.183845429	1.89767E-05
GP2	2.180619488	0.017834276
DARC	2.163632861	8.45267E-11

SSTR1	2.161870812	0.00419041
OXTR	2.150387525	0.000114189
CARMN	2.148274249	1.56758E-09
SLC14A1	2.148046851	0.019976688
PYDC1	2.14773931	0.002505426
CDH20	2.146896394	4.98415E-05
UMOD	2.143085079	0.000119803
ZBTB16	2.142899088	3.09925E-05
DLX2	2.141589051	0.00139153
SLC1A1	2.134904785	2.34401E-05
SCN7A	2.134410878	4.78668E-08
SCN2B	2.132955293	2.74488E-09
LCN10	2.131713118	0.003042708
KCNA1	2.130988187	0.000867263
SLC26A5	2.128189454	2.81657E-06
CIDEC	2.127218862	8.81386E-05
NEK10	2.12584836	3.88655E-09
SGCA	2.121865627	0.004271255
TNXB	2.114644829	6.61491E-10
FAM196B	2.112059721	0.013002919
SHROOM1	2.108625919	1.57244E-06
GRIA4	2.108386585	0.006080956
FGF10	2.103119426	0.029708617
SRL	2.101930923	0.002838761
CACNB2	2.090727145	0.014221225
C2orf73	2.085637672	8.17834E-08
CYP4X1	2.082874264	6.8775E-07
SLC6A4	2.073442717	0.017241094
CLEC17A	2.072342844	0.018500949
SORCS1	2.070192175	0.000355623
AQP7P1	2.065551703	0.000410275
FCGBP	2.063003289	2.16647E-05
HBB	2.057950809	0.000199876
FAM180B	2.053380077	0.000589928
TRHDE-AS1	2.052965321	0.004633239
CDH22	2.044671665	0.028608839
LEP	2.040471613	0.005644831
CACNA2D3	2.038840327	0.002853203
ANGPTL1	2.038629565	1.68317E-09
C9orf68	2.037050988	9.17247E-14

SERPINA11	2.035253614	8.74941E-07
C10orf107	2.032939216	2.51369E-05
GALNT5	2.031427692	0.002343196
NTRK3	2.02132064	0.003842384
ADRA1A	2.016207639	0.001390121
CYP4A11	2.015127567	0.034985304
IGFALS	2.014087092	8.23325E-05
ADH1B	2.011322017	0.000111455
AJAP1	2.008582344	0.043680166
CASQ2	2.007719841	2.1818E-05
EFCAB1	2.003565549	0.018682497
LRP2	1.999337886	0.000458985
FREM1	1.998234354	5.44504E-09
NAT1	1.997172267	6.75176E-07
GPD1	1.994584616	0.000110343
CCL14	1.989179411	4.85574E-10
KRT8P41	1.988979448	3.25957E-06
PLIN1	1.983916435	7.22328E-05
HSPB7	1.978152077	0.000109404
ADAM33	1.978136017	5.87217E-13
ANGPT4	1.972643602	1.15329E-05
HPSE2	1.969304448	0.040291905
SPATA4	1.96743412	1.53087E-08
SLC22A11	1.96630769	2.98398E-05
ZNF471	1.960021908	1.87955E-26
MAP1LC3C	1.95762162	2.15467E-05
IL1RL1	1.957000308	5.23237E-05
NTN4	1.955538008	9.1046E-17
GPIHBP1	1.948961302	2.30655E-11
SCN4B	1.948872003	1.64111E-15
ABCB5	1.948831811	0.002296421
CACNA1F	1.946082794	3.14446E-07
KCNE1	1.944932548	0.000106133
C8orf79	1.940224085	6.32358E-12
TPO	1.937338133	7.30278E-09
ZNF677	1.933273828	3.47878E-19
RD3	1.930193074	0.000994203
ADAMTS8	1.92728786	0.002953493
MFAP4	1.925469856	1.12379E-12
LTF	1.924948128	0.005299654

CCL21	1.923370661	0.000468221
PI16	1.920953063	0.002753447
NPY1R	1.92041721	0.016324627
COL4A6	1.919294378	0.006273563
CNN1	1.915434054	9.6944E-12
NXNL2	1.913168904	2.34746E-07
C8orf84	1.911381596	6.35362E-05
CIDEA	1.910941253	0.00814828
PAMR1	1.910020126	1.12404E-11
PTPRT	1.908880149	4.71354E-08
ADIPOQ	1.908779985	0.00037854
B3GALT1	1.908345335	0.002398414
CYBRD1	1.905798242	7.02396E-14
KCNIP2	1.905042834	1.83884E-05
GRM4	1.902757929	0.000461249
EGR1	1.897054364	1.6987E-09
FOS	1.895886117	1.28078E-08
LRRC48	1.890461683	4.90091E-11
GATA1	1.889227237	3.09378E-06
PGM5	1.886945227	2.68442E-07
TMEM26	1.884732551	2.95111E-07
PLAC9	1.882251798	8.58261E-11
CLEC9A	1.881028379	1.32195E-07
IRX2	1.880037398	7.15308E-08
MASP1	1.877602738	0.005773288
EGR3	1.876774054	1.43467E-07
SIGLEC15	1.872901686	0.000423145
MYOM1	1.872548571	0.003142649
NCRNA00093	1.870445382	3.72555E-07
SELP	1.869836443	9.49757E-10
AVPR2	1.867689923	2.46385E-10
IL33	1.867600979	1.57547E-07
SCGB1D2	1.862763133	0.016565341
TPSB2	1.85392651	6.86324E-06
CBLN4	1.851797001	1.58917E-09
KCNQ5	1.851241271	0.000615349
ABCA9	1.850538991	2.40634E-09
CD1C	1.849273087	2.31796E-07
TP63	1.846158888	1.3983E-06
CDO1	1.846019646	4.17746E-07

MYOCD	1.845482476	0.028748947
CCL19	1.843873383	0.001833539
MIR205HG	1.842038906	5.21351E-06
MMRN1	1.841180322	4.62269E-06
ITIH3	1.841137701	6.68782E-05
BMX	1.840788974	2.47604E-08
HEPACAM2	1.838233591	0.006776373
C6	1.837742478	0.005384014
ATOH8	1.837696014	3.09933E-08
CX3CR1	1.837279263	6.7835E-09
TPSAB1	1.837240534	1.6046E-07
AQP7	1.836352328	0.000530037
TACR1	1.832858449	0.000258548
CYP4Z2P	1.832828478	2.39259E-05
CCDC65	1.83254029	0.000773206
MUSTN1	1.830959569	0.000117686
PLAT	1.830017596	0.001535571
RASGRP2	1.829125294	0.00670412
COL14A1	1.827649828	1.70506E-09
DNASE1L3	1.824731622	4.62518E-05
MAPT-AS1	1.823556624	5.71567E-05
CDHR3	1.823458564	0.023352691
CAPN8	1.819305801	4.06038E-07
TMEM47	1.819226729	0.000490965
TPSD1	1.816792342	0.041160788
HSD17B13	1.815349724	0.000189561
TRIM63	1.807962091	0.047329253
ROBO2	1.80742478	0.014551634
SCUBE1	1.806316948	0.004010804
GIPC2	1.806181623	2.74488E-09
NOSTRIN	1.806081128	6.28047E-15
CDH12	1.80568275	0.005097352
ZNF542	1.80503195	1.7483E-48
LINC00926	1.804956653	0.022006404
ZNF238	1.803253501	5.7224E-09
LRRC2	1.802283934	1.93131E-06
KIF13B	1.800983578	2.10312E-18
PTCH2	1.800092019	1.6987E-09
INMT	1.798589352	8.40966E-11
TSPAN7	1.796964065	5.63941E-12

MAMDC2	1.796661687	5.88185E-08
SIGLEC6	1.795670373	3.17363E-06
PDK4	1.79012926	2.71646E-06
CLDN5	1.787827778	4.6246E-11
CCDC74A	1.786252069	0.000301179
PM20D1	1.785578098	2.1173E-06
DBX2	1.785383164	1.23398E-08
ZNF154	1.780223966	4.29152E-09
SAMD5	1.779634496	0.000251204
SIGLECP3	1.777097355	7.54602E-09
PCK1	1.774724077	0.003984067
GRIK5	1.774714683	0.000432239
CHRDL1	1.77343003	5.99806E-06
FHL5	1.773414558	2.24259E-07
DNASE2B	1.771201605	0.012313682
MS4A2	1.771156804	5.61672E-07
SLC8A3	1.770638368	2.05689E-07
PHYHD1	1.769629429	1.59257E-12
PDZK1	1.768944032	0.003139236
C4A	1.765834272	7.03984E-10
C11orf53	1.761073246	0.005760935
ZNF667	1.758845481	1.7306E-13
THSD7B	1.758083103	1.04337E-07
ITGA7	1.756702309	1.09026E-07
RGS13	1.749389558	0.00042431
ABCB1	1.74847205	1.08701E-10
LRRC3B	1.748392106	0.028427358
SYT9	1.747453769	1.12627E-06
NCAM1	1.746120449	0.019019719
C4orf31	1.746044553	0.001333151
ENTPD5	1.74390431	0.000910419
LRRN4CL	1.742215124	1.62127E-10
KLF15	1.740670304	1.73468E-05
SUSD3	1.738550408	1.27648E-08
TFF1	1.737407636	0.010935488
ELN	1.736137484	0.000305782
IGF1	1.733355091	8.16726E-09
FST	1.731619092	0.015983361
PDZK1P1	1.723814092	0.018727159
TPRG1	1.721326261	0.002354847

TP53AIP1	1.71903048	2.2619E-05
ATRNL1	1.717846301	0.047598838
HBA2	1.715735569	0.00050114
NHLRC4	1.714087266	4.81006E-07
FAM81B	1.712925789	0.033472725
KEL	1.712113868	0.008792575
RTN1	1.709886155	2.04652E-05
TMEM232	1.709300095	0.000143212
ST8SIA6	1.709274357	6.472E-10
BTNL9	1.706104351	1.32273E-08
ANKRD43	1.705969513	0.000665373
FGD3	1.703196341	4.58982E-08
WDR65	1.701673999	4.2349E-07
CACNA1D	1.699331034	3.66822E-11
RAI2	1.699047138	6.91836E-11
CD1E	1.696859186	5.52416E-05
NGFR	1.693001891	1.73658E-05
NLGN1	1.692640463	0.019669453
CCR6	1.692237035	0.008857912
KCND3	1.692234432	2.63698E-06
PSD3	1.688943308	2.13092E-07
HLA-DQB2	1.687789198	0.001811646
C7orf63	1.686645526	1.42151E-11
HSPB6	1.686561664	0.000113191
IGSF21	1.685770685	0.000239607
ITIH5	1.684935137	1.39943E-07
HOXA2	1.684604959	0.000490457
ABAT	1.683904938	4.68318E-10
APOF	1.683756122	0.001526721
NEFL	1.68261153	0.003094823
GP1BA	1.681091852	0.012706005
F10	1.678454817	7.23656E-11
ZIK1	1.678158323	1.0027E-05
HOXA5	1.677657458	0.00062012
HLF	1.67687971	5.17746E-05
PLD4	1.676259466	5.54447E-10
MAB21L1	1.675628565	2.83119E-09
CLDN11	1.675285289	4.52722E-07
C1orf150	1.674137205	0.00060033
FGF2	1.671525416	0.001852354

DLEC1	1.669967114	0.000266579
ZSCAN1	1.668827856	6.10483E-06
GJA1	1.668362117	0.000474089
C6orf217	1.668344131	4.20018E-08
NEFM	1.666783617	0.027069861
CFP	1.665611493	7.55557E-05
SCG5	1.65628932	0.020752145
UBXN10	1.65594222	1.42151E-11
DLK2	1.655257675	0.000409259
P2RY12	1.653428737	1.64378E-07
HOXA7	1.650587238	0.030034755
PLIN4	1.649341543	0.009886519
CYP21A2	1.645772968	1.56871E-06
MAGI2	1.644608435	0.00354302
GLI1	1.643103697	2.9149E-07
LOC93429	1.642730395	0.034593512
TUSC5	1.642640894	0.040620048
MAK	1.641750651	2.15866E-05
GRRP1	1.640907975	1.38626E-09
LMOD1	1.640636001	9.66379E-11
STK32B	1.637459936	1.28045E-05
MYOM3	1.635023063	0.016662352
TSLP	1.634807672	0.000763558
PTPN5	1.631627421	0.000978002
LTC4S	1.626535163	9.6944E-12
LINC00965	1.626519603	7.88078E-10
SPARCL1	1.626101258	1.025E-15
CPA3	1.625974263	8.90795E-05
SLC40A1	1.621857339	1.06494E-05
C15orf51	1.621728665	5.08929E-08
ZFP36	1.621453703	3.63221E-08
TMEM84	1.621286319	0.000274558
GEM	1.620927888	1.69583E-09
ADAMTS19	1.620259137	0.010131919
CD22	1.619987066	0.009237106
TNN	1.619884352	4.56971E-06
RHOXF1	1.619706744	0.003528083
PRAG1	1.616889253	5.23393E-12
EGR2	1.616679416	1.2649E-06
PRKACB	1.614624521	0.018387599

SETBP1	1.613035619	1.38619E-14
LIPE	1.612930787	0.000124066
ENPP5	1.612848709	5.11144E-05
C21orf15	1.612309904	0.013253566
VWA2	1.60894536	1.62041E-08
FANK1	1.608675905	9.69077E-12
ASPA	1.607611641	1.00245E-06
AQP1	1.606539536	8.59856E-11
ABCA6	1.605385029	4.49553E-09
RDH5	1.604457346	0.00075918
FLJ42875	1.603856105	0.005278587
C18orf34	1.603847163	0.028947453
TSSK1B	1.603466253	1.2879E-07
FAM13C	1.603303007	1.8052E-10
COL6A6	1.599931693	0.002479424
VSIG2	1.599473298	0.022615131
DNAH7	1.599084452	4.4689E-08
PREX2	1.59787086	2.17431E-08
KIT	1.597235358	0.001911511
HPGDS	1.596037912	1.2732E-11
NCRNA00086	1.594858818	1.25021E-05
HOXA6	1.594552493	0.004381173
PTGDS	1.593925603	0.003409654
PDGFD	1.591306424	2.75896E-14
FRMD1	1.589429237	0.001866161
ZNF835	1.589169567	1.16775E-08
FABP4	1.587989217	0.016427314
TTC16	1.587467386	7.77568E-05
SCN4A	1.585789384	2.8631E-06
ITGB3	1.585692205	0.040430963
C19orf36	1.58555242	1.26052E-09
FHL1	1.585266099	9.69461E-05
FAM162B	1.584267519	2.21089E-11
ATP1B2	1.58391666	1.94505E-08
PDZRN3	1.583537467	1.0792E-10
TLR10	1.583303138	0.010879394
PDE1C	1.580947404	0.041072453
PON3	1.580923621	0.020496956
KCNS2	1.580349021	2.15731E-05
LOC100129534	1.580257528	3.12312E-14

PER1	1.580080705	1.68317E-09
CSRNP3	1.579530823	0.000143192
DPT	1.579042235	4.16272E-07
WNT9B	1.577708741	1.06043E-07
ASAH1	1.577453245	7.03637E-09
CASC1	1.576173735	4.22959E-06
PRICKLE4	1.572566772	5.67877E-09
GLIPR1L2	1.572463681	2.25047E-07
PELI2	1.57209653	1.0792E-10
DCDC1	1.569331849	5.22544E-05
LINC02731	1.569141119	0.001579467
ZNF671	1.567171352	3.16525E-11
ADCYAP1R1	1.566258922	0.011389667
ZNF578	1.56584833	1.56994E-07
KLHL29	1.565470654	4.98267E-06
CDK15	1.564883603	7.97428E-06
GRP	1.564490593	5.65549E-06
EBF2	1.563181008	1.00692E-08
AOC3	1.563121474	2.25836E-07
IQGAP2	1.563120357	7.54602E-09
USHBP1	1.562269305	8.5277E-12
DYNC2H1	1.561965406	2.7523E-12
NGF	1.56131804	0.037965645
ASTN1	1.561294826	0.03663488
LAMA2	1.560710736	1.35045E-09
SLCO1C1	1.560595189	1.14127E-07
RSPO3	1.559221946	0.009830299
TGFBR3	1.558692527	1.34535E-05
ADAMTS15	1.558033371	3.43182E-05
CA14	1.555809654	0.020147228
MMP23B	1.555445781	4.03167E-07
GPRASP1	1.555149057	1.18007E-10
SYT6	1.555090553	0.024850572
EFCAB12	1.553087616	2.75266E-06
NCRNA00173	1.552962456	0.001376417
SVEP1	1.552731383	6.06007E-09
CSDC2	1.551209499	2.618E-07
ZNF502	1.550737929	2.87898E-14
ZNF540	1.550225283	2.28236E-13
HCG22	1.549562553	4.39992E-05

ZNF214	1.549150259	5.72977E-12
DMD	1.548451127	0.006477909
DCLK1	1.548402052	0.012665361
S1PR1	1.548203592	1.87089E-11
ECT2L	1.547961091	0.017945417
SLC19A3	1.547772574	0.01751402
PCDP1	1.547019219	0.039750344
MST1	1.546917235	8.06392E-09
SPAG17	1.546262776	0.025941309
RNF183	1.544819683	0.006679643
GALNTL2	1.543390092	3.28618E-06
SSC5D	1.543384867	1.0783E-08
LEPR	1.543285198	4.33145E-08
FAM189A2	1.543016018	3.23409E-06
C1QTNF7	1.542977304	0.000844235
OR2W3	1.542638591	0.000277164
ZNF577	1.541649962	2.45813E-15
CRB2	1.541313066	0.000301179
NPR1	1.541226784	3.94214E-09
SLC5A4	1.540676234	2.27719E-06
RASSF6	1.539930255	0.021119554
CAV1	1.539447144	2.30655E-11
LGI4	1.537451402	4.57626E-07
FAM159A	1.537391418	0.000138088
CYR61	1.536635489	4.43384E-08
MRGPRF	1.534301101	2.39745E-09
STX1B	1.533679505	3.43609E-08
DHH	1.533270915	1.90748E-07
FAM182A	1.533187453	0.000869668
RAPGEF3	1.532211388	4.73345E-15
OR52N4	1.531808906	0.006020229
CRB1	1.531035794	0.002529505
CYP2B7P1	1.52997645	0.017566469
ANKRD24	1.529383748	1.81984E-05
ELMOD1	1.527737545	0.008485267
PHYHIP	1.527474006	0.002495215
GIPR	1.527329309	2.34042E-05
BHMT2	1.526826374	6.13545E-05
ZNF300P1	1.526407395	6.61211E-08
PODN	1.525411481	1.54022E-10

SEC14L2	1.524418442	0.00013815
LOC283174	1.524348173	0.000881888
AASS	1.524113418	4.67265E-15
EMCN	1.521806288	5.63972E-12
STC2	1.521176326	0.001780703
MACROD2	1.521122886	0.037008408
PGR	1.52048811	0.004140232
PIK3C2G	1.520331264	0.028344557
DUSP1	1.519886026	1.67283E-07
SLC17A7	1.519252394	6.27292E-06
SPATA18	1.517771366	1.99777E-06
LRRC70	1.517441965	1.28077E-11
CLUL1	1.516689318	6.46991E-05
SLC43A1	1.515007982	7.7771E-08
CLEC3B	1.514390469	1.58389E-06
PRR16	1.514132366	0.001840265
EDNRB	1.513724022	1.54731E-08
TRIM74	1.513342029	0.002586479
LRRC50	1.513230688	0.000239023
NOVA1	1.512922376	0.013061829
OSR1	1.512706025	0.033147734
FAM149A	1.511543197	1.04289E-07
CCDC141	1.51141884	0.010429079
FAM38B	1.510415397	0.004485714
DNAH6	1.509612711	0.008474531
PDE2A	1.508786675	5.60345E-08
BAI2	1.50747465	1.94247E-05
SLC5A9	1.506877079	0.003163708
TM4SF18	1.506795395	0.000404925
KLHL13	1.5067507	0.002165388
CH25H	1.50587258	8.06246E-06
C10orf93	1.505109904	0.00016283
CLEC10A	1.50469832	0.000734382
NRIP2	1.502892869	1.1317E-15
SPEF1	1.50219601	5.71338E-05
CBX7	1.501583976	6.4328E-16
LINC00924	1.501171532	1.19318E-06
FAM66A	1.500804635	1.95626E-05
TCEAL7	1.500624753	1.70331E-06
EEF1DP3	1.500550194	5.61496E-05

LHFP	1.500314311	9.25857E-15
MKI67	0.666414739	1.66499E-11
PPFIA1	0.6658262	5.95011E-08
FAM83B	0.665751832	0.000858882
PFKP	0.665639017	3.55635E-06
PRC1	0.665292774	2.13226E-11
OIP5	0.665043386	3.64408E-14
KIF4B	0.664982044	4.62001E-13
ESPL1	0.664772354	2.06591E-12
CLIC3	0.664026392	0.014215402
HTR1D	0.662802824	0.005452098
ERCC6L	0.662437059	6.81032E-14
C3orf57	0.662354683	0.014113462
SMIM5	0.662034004	0.001575874
FAM72A	0.661712102	1.20201E-07
ORC1L	0.660957927	2.86603E-08
FAM171A2	0.660708536	0.000239225
CDCA5	0.660609174	3.55825E-09
NEIL3	0.660523693	2.37392E-10
P2RY6	0.660430209	6.7355E-05
GRB7	0.660333023	0.012425979
EIF4EBP1	0.660167702	6.567E-05
PTTG3P	0.659985537	1.82949E-05
KIF4A	0.659523576	1.4424E-13
E2F5	0.659375844	4.67472E-11
ACOT11	0.65914577	0.001373509
CDC25A	0.658438889	7.84386E-09
RHBG	0.658213059	0.018068089
ORC6L	0.657773187	2.97196E-09
ME1	0.657401139	0.000466688
KIAA1524	0.657400143	7.81969E-11
CENPA	0.656776627	2.53369E-07
FOXC1	0.656556977	0.024085608
DEPDC1B	0.656527043	4.82146E-11
MND1	0.656503009	2.03272E-07
CDKL2	0.655929716	0.010813973
NCAPG	0.655536526	2.9394E-13
TK1	0.655190574	1.92853E-06
NCAPH	0.654944624	4.83496E-13
UBE2C	0.654191843	2.36428E-08

CDC20	0.653860927	1.13328E-07
KIF2C	0.653546114	7.47401E-09
HPSE	0.65327069	3.76899E-09
CDC6	0.652878257	0.005283417
SKA1	0.652468098	1.05798E-08
CDKN3	0.652388152	4.25514E-11
NDC80	0.651771458	8.77692E-10
MAD2L1	0.651495067	3.535E-13
FA2H	0.651287218	0.005006416
CKMT1A	0.651245878	0.000144495
C17orf96	0.650949469	0.010640173
SPTBN2	0.650159629	0.035930712
NAP1L6	0.650126319	0.038178216
CD24	0.650099881	0.000738362
RAET1G	0.649424949	0.00678137
FANCA	0.649206355	1.43856E-08
CCNE2	0.647499538	1.0344E-07
NME1	0.646648077	1.32886E-05
E2F2	0.646207524	4.1258E-08
GTSE1	0.646134727	1.3284E-11
MYBL2	0.645888536	4.74623E-06
ELOVL4	0.645781541	0.049164791
PSMD12	0.645738912	7.06021E-18
GBP5	0.645479021	0.010364701
BLM	0.645476844	6.46808E-08
ARHGAP11B	0.645118177	9.27031E-11
BMS1P20	0.644939801	0.025100722
HIST1H3B	0.644922135	0.022232376
MED1	0.644895411	0.000814179
SCD	0.644367541	3.11013E-06
TMEM171	0.643687633	0.036044982
AURKB	0.643639229	1.19011E-08
HCN2	0.643415711	0.033879839
SLC16A10	0.642982914	0.002101803
RAD51	0.642909879	9.1046E-17
EME1	0.642809755	2.56893E-06
IFI27	0.642397751	0.000558246
TMEM52	0.64237577	0.000135218
HJURP	0.642187651	6.5228E-13
BDKRB1	0.64190349	6.97988E-07

ZNF695	0.641476328	2.11829E-05
GSG2	0.641215517	1.90586E-13
CENPW	0.640850514	0.001378174
SOX11	0.64054832	0.018318047
KIF18B	0.640547239	2.02327E-10
HIST1H2AJ	0.640382836	0.000674181
LILRA3	0.640378945	0.001446057
IMPA2	0.640310935	4.41665E-06
MELK	0.639859854	8.37617E-10
FPR2	0.639354312	0.007708924
HS6ST3	0.639244353	0.006872888
ELOVL3	0.638696859	0.012282864
LAD1	0.638526558	8.15265E-07
SV2A	0.638521319	0.003721676
PLCH1	0.637373683	2.05987E-05
IL8	0.635767978	0.024664267
CLTC	0.635011799	5.05792E-09
SMC1B	0.63417214	0.022663153
CDC45	0.633910447	3.19582E-12
ULBP3	0.633500802	0.001486232
SKA3	0.633307049	6.32254E-11
DLGAP5	0.633102842	6.22764E-13
USP32	0.631984667	8.3924E-07
XK	0.631639484	0.000481269
CENPE	0.631596413	1.38619E-14
B3GNT3	0.63152681	0.004878131
CELSR3	0.630854965	1.57963E-06
MYBL1	0.630517398	9.29928E-05
SHCBP1	0.62968796	7.57507E-15
POLQ	0.629673334	3.36615E-14
CCDC144C	0.629043982	0.02581364
ITGA2B	0.628850796	0.002717094
TPX2	0.628413688	8.35857E-14
TOP2A	0.628201953	7.57946E-07
HR	0.628019605	0.000108969
RAPGEFL1	0.627942859	0.008560007
CEP55	0.627443817	1.11195E-11
CCNB2	0.626505003	6.89226E-13
CLSPN	0.626409632	7.62914E-11
TBX1	0.62638512	2.09138E-05

ARHGAP11A	0.626295864	3.38311E-16
CENPI	0.62613643	5.67182E-14
HIST1H1B	0.625553375	0.004564879
APOBEC3A	0.625483922	0.000769237
BUB1B	0.624545267	3.34744E-18
PIR	0.624388185	2.5791E-06
NOXO1	0.624144491	0.015575638
CASC5	0.623610975	1.40264E-14
PLK1	0.623560908	1.4424E-13
PANX2	0.623104422	9.50049E-05
CP	0.622391304	0.026292479
KCNG3	0.622066049	0.000310915
ERBB2	0.620819729	0.005328513
RRM2	0.619945991	1.57218E-11
SLC9A2	0.619182351	0.000617688
SPAG5	0.618184114	1.4486E-11
NPR3	0.61699767	0.008286453
PADI2	0.616195917	0.000395357
ARX	0.61569299	0.038411641
DIAPH3	0.61514258	9.51108E-09
ASPM	0.615012079	3.8969E-13
CHAC1	0.614892141	7.4913E-06
KIAA0319	0.614609877	0.018359424
ANLN	0.61422049	1.57218E-11
TMEM45A	0.613766614	0.001322567
GPR156	0.613466247	0.006567929
SGOL1	0.612804546	7.08961E-13
CKAP2L	0.612335234	3.96962E-16
CXCL11	0.610740295	0.001389152
LRRC69	0.609707076	0.002925066
BUB1	0.60910634	1.38462E-13
PROM1	0.608565377	0.002101803
GPR158	0.608305647	0.016662352
PLAC2	0.608195309	9.09515E-06
CDCA2	0.605433137	6.1966E-07
DEPDC1	0.604428053	9.72249E-10
MAP2	0.603784096	0.002433447
HIST1H2AL	0.603772761	7.69941E-05
CBS	0.603237144	3.60018E-06
SQLE	0.603041454	3.9016E-13

EXO1	0.602990889	1.15103E-13
TTK	0.602237767	2.70598E-11
EN1	0.601311235	0.0226572
FAM54A	0.599290416	1.32323E-11
CEACAM5	0.598121238	0.049432826
BRIP1	0.597498608	1.30029E-12
PSAT1	0.596966874	0.002859182
MKRN3	0.596202562	0.000279303
CLDN1	0.595434731	0.017413132
KIF14	0.594292898	3.86443E-13
GUCY1B2	0.593284023	0.000266614
FBXW10	0.593273706	0.001759547
KIAA1751	0.592265217	0.030257394
CDCA7	0.592030804	6.17107E-06
RAET1K	0.589660507	2.02252E-05
CTSL2	0.589095648	0.003051175
AURKA	0.588677125	1.96393E-14
SLC22A20	0.58835881	0.007649737
CKMT1B	0.588089493	7.90334E-07
DSCC1	0.587963697	5.18818E-16
PAH	0.587095477	0.025897665
CCNA2	0.584900076	6.48242E-16
CAPS	0.584691758	1.60903E-05
TRPV3	0.584627959	0.015385712
GBP6	0.583456921	0.040252086
DLL3	0.583195147	0.039603794
GJB3	0.582705232	0.038997716
ATP1A3	0.58212495	0.037496038
CYP3A5	0.581430702	0.018756794
FAM131C	0.579497291	0.001972238
EPR1	0.578560851	4.12549E-10
B4GALNT1	0.578175586	0.000481494
MMP7	0.577848114	0.04595728
DKK1	0.577205695	0.027415169
C15orf42	0.576365673	2.27681E-09
CXCL10	0.575305335	0.001043204
DQX1	0.575152044	0.000250167
BIRC5	0.575099483	4.24004E-10
RASGRF1	0.574829779	0.002348011
MUC15	0.573158417	0.01824779

SLITRK5	0.571444597	0.009798576
FABP5	0.570002205	0.007588501
FAM83D	0.569333127	1.02853E-13
PKP1	0.568873923	0.011932785
RDH10	0.568708785	0.008012465
SLC6A11	0.56847873	0.000530037
C21orf125	0.568085307	0.000954634
ZP1	0.567528243	0.04955445
NXPH4	0.566464619	0.006623503
LHX2	0.565738806	0.015818693
CBX2	0.565582137	8.56303E-09
IGF2BP2	0.56466909	0.003501067
PNPLA3	0.563656622	0.001804913
LRP8	0.563331334	2.02884E-11
HPDL	0.561573671	0.0004622
COCH	0.559769549	0.003836217
GDPD3	0.559652804	0.017900837
E2F8	0.559153567	6.32358E-12
ELF5	0.559104084	0.011216282
SLC28A1	0.55903159	0.042934126
DMBX1	0.557485778	0.000304479
KPNA2	0.555604444	6.95437E-24
ALG1L	0.554338165	2.96907E-05
AQP9	0.553069097	1.6242E-05
PCP4L1	0.553041296	0.040984971
SLC17A9	0.552744245	3.47976E-05
TDO2	0.55147354	0.001106851
GPR126	0.550747593	1.33898E-07
ZIC2	0.550073208	5.00792E-05
DHRS2	0.548268425	0.038444322
CCL8	0.548117182	3.61264E-05
RGS20	0.547278898	0.027544533
CYP4F3	0.544640202	0.02459809
CCNE1	0.542512439	0.001552429
SFTPA2	0.542499823	0.010790129
CHRNB4	0.542462197	0.016601633
ULBP2	0.540712599	5.73383E-05
SLC7A5	0.538494396	6.88866E-06
FOXD1	0.537414849	0.000763028
MMP1	0.537149413	0.005192649

CECR2	0.534830305	6.4744E-07
DLX6	0.534217048	0.047580239
DNAH11	0.53333688	0.018602457
PPARGC1A	0.533142671	0.033895502
C17orf104	0.532713312	0.004198721
IGF2BP3	0.532548603	0.037759328
DLX4	0.530933498	2.67981E-06
CHODL	0.530300394	0.01482932
KLRG2	0.526327445	7.8462E-07
CHRNA5	0.525972258	8.1936E-10
C10orf91	0.52573331	0.004111597
CALML5	0.524557336	0.003269837
MCM10	0.523821527	7.29972E-13
B3GNT7	0.521992642	0.008926533
S100P	0.519798162	0.000366336
PPP1R14C	0.519545017	0.004185692
KLK8	0.518046455	0.037327797
RSPO4	0.514658231	0.008084398
C1orf106	0.513758093	5.18668E-08
NPW	0.513609887	0.032828698
TMEM64	0.511853785	0.010790129
GABRP	0.510652932	0.001886991
NCCRP1	0.510065494	0.011714262
TPRXL	0.508608563	0.00908553
DDN	0.503427355	0.012274237
CWH43	0.499020837	0.03008553
ATP6V0A4	0.497946763	0.000160877
MUC16	0.496719106	0.003747254
APOBEC3B	0.49610433	1.35042E-07
COL22A1	0.495702074	0.010465089
ACSL6	0.495688212	0.006378147
LCTL	0.494020643	0.036391357
GPX2	0.484811614	0.011283202
GAL	0.484565166	0.001834288
TMPRSS4	0.481851555	0.008189799
ULBP1	0.477530208	6.55888E-07
CRLF1	0.474151521	0.041734654
BAIAP2L2	0.466694935	0.001818486
IYD	0.460560304	0.006896998
ANKRD34B	0.456566071	0.021544488

KLHDC7B	0.455354452	0.014634128
HOXB9	0.45512983	0.033351083
PGBD5	0.453391254	0.000207171
ONECUT2	0.452636304	0.000125697
SLC6A15	0.440461911	0.03716639
DIRAS2	0.436743233	0.01997368
LCT	0.433733249	0.002838823
VGLL1	0.431153733	0.026821714
SERPINB2	0.42780741	0.047026575
TNIP3	0.427409192	0.012204143
MT1H	0.422777755	0.008375341
DMRTA1	0.417911816	0.000214342
SLC6A14	0.41701213	0.044514944
FAM83A	0.402966829	0.002841903
PRAME	0.392253199	2.30197E-06
C1orf186	0.386453771	0.008474352
GSDMC	0.384519764	0.00021888
WNT3A	0.37604418	0.009895588
RASGEF1C	0.374268838	0.008272486
CEACAM7	0.370393775	0.049969295
DUSP9	0.370278783	0.000350862
DSG3	0.370048039	0.002450702
KRT16	0.364673838	0.015146936
PRR11	0.361882564	0.01003788
KCNG1	0.358043108	0.008816362
WDR72	0.356320604	0.030275519
KCNK9	0.353467219	0.01629245
GDPD2	0.352253034	0.013169055
MATN4	0.348927417	0.042229763
AKR1B10	0.346289863	0.005238294
KRT81	0.345064452	0.001985938
CCL7	0.340600948	5.38949E-06
SLC15A1	0.32382801	0.039864341
CELF3	0.293745567	0.037799841
C6orf15	0.289811431	0.021119554
CHD5	0.28924947	0.034602012
NRAD1	0.28904838	0.004749939
ABP1	0.277372914	0.014086818
PSORS1C2	0.254593402	0.02995255
LASS3	0.237050096	0.043958065

ACTL8	0.177612122	0.000490876
CRISP3	0.104141806	0.007507755
MAGEA8	0.097125143	0.044307011

Supplementary Table S3. Genes in the *ZNF582-AS1* methylation signature

Gene symbol	Fold change	Adjusted p value
DLX4	2.001458112	0.0169723
CHRNA5	1.731670233	0.007555658
B4GALNT1	1.642542881	0.042161372
ETV4	1.630874315	0.022127598
PIF1	1.605762561	0.039508544
CNIH2	1.600911277	0.042740036
CBS	1.578019275	0.044556023
SLC26A10	1.544203015	0.03292953
EPR1	1.521123884	0.006982047
CELSR3	1.510186313	0.033927392
BDKRB1	1.500947723	0.014318591
FAM81A	1.481057253	0.041407244
CCNE2	1.473889553	0.009457659
MURC	1.4698396	0.021028916
CDKN2BAS	1.468679137	0.044556023
ACCN2	1.464247741	0.02448453
BRIP1	1.456852392	0.011039893
UHRF1	1.446663456	0.001130333
CDCA5	1.429790104	0.019923311
MELK	1.420846512	0.017592968
EME1	1.417283659	0.035429907
C18orf56	1.415154922	0.027428984
TYMS	1.413976474	0.014340796
C15orf42	1.413298763	0.028702757
CDC7	1.409143594	0.006582365
SPAG5	1.408975299	0.011039893
SKA3	1.402221556	0.025292164
ORAOV1	1.400352449	0.025486382
CDT1	1.396174833	0.011936026
RAD54L	1.389923602	0.021028916
AURKB	1.380627053	0.049882389
CDKN3	1.370621975	0.027772811
PTRH2	1.370432623	0.00756483
GTSE1	1.366722388	0.013693698
RHEBL1	1.36588215	0.011936026
GINS2	1.358506543	0.014318591
KIF2C	1.355911605	0.037526789
CHAF1B	1.355646503	0.00425459
E2F1	1.354865883	0.017592968
RRM2	1.353620028	0.045660908

CDC45	1.351394224	0.0169723
EZH2	1.346944149	0.023564678
KIF18B	1.343984538	0.035327167
KPNA2	1.341763296	0.008257094
TUBD1	1.335573034	0.006582365
CCNB2	1.333186692	0.019923311
DDIT3	1.330865171	0.0169723
WDR62	1.323962633	0.027653933
PPP2R3B	1.323798822	0.011690184
TMEM49	1.323475268	0.018809447
SKA2	1.318208002	0.026454098
DONSON	1.317851377	0.008257094
PLCXD1	1.316027106	0.021028916
PPM1D	1.313083996	0.048131804
OIP5	1.303406848	0.017592968
TYRO3	1.30229723	0.013693698
KIF4A	1.301172003	0.029954845
CLTC	1.299102131	0.038305741
THOC4	1.297726982	0.011039893
RAD51	1.29733777	0.013080367
HJURP	1.295305415	0.040649611
CENPN	1.294078017	0.038305741
PSMD12	1.294032288	0.003994498
CENPK	1.29177347	0.029954845
POLQ	1.288408105	0.048227955
DDX39	1.287687293	0.006982047
NCAPG	1.28699036	0.035429907
MCM2	1.285510301	0.028490482
CENPE	1.284188441	0.045766351
MLF1IP	1.276600908	0.014383982
BUB1B	1.27218722	0.033359675
CTPS	1.269524362	0.037526789
METTL2A	1.269346924	0.004323018
RAD51C	1.265567282	0.014997385
C8orf38	1.25784278	0.040649611
NUP85	1.256531393	0.001055254
INTS2	1.254462217	0.03292953
NOL11	1.25411105	0.00301687
ASF1B	1.252018254	0.036480861
GALK1	1.250534694	0.014383982
MYO19	1.249278676	0.021028916
RPS6KB2	1.247425635	0.013693698
SNRPA1	1.247319187	0.019564492

CYB561	1.243636954	0.043915596
TACO1	1.241467993	0.008257094
SLC25A19	1.236452437	0.019332462
SLC4A5	1.236323454	0.011608521
TRIM37	1.235406661	0.043100118
C16orf61	1.235352686	0.01776313
MCM6	1.233988347	0.014997385
CHAF1A	1.232784883	0.01928333
PSMC5	1.232762769	0.044176381
DHX40	1.229154475	0.045660908
IDI2	1.228807768	0.021802063
CCDC45	1.226933228	0.011039893
LMNB1	1.226741754	0.038809852
ECT2	1.225403274	0.041407244
ICT1	1.224463674	0.044556023
WDHD1	1.222537625	0.041407244
ZCCHC11	1.222424725	0.038809852
FANCL	1.221342157	0.042538282
AMZ2P1	1.219487853	0.029954845
FAM100B	1.218296287	0.040314929
AMZ2	1.216395576	0.007857713
TLK2	1.215304028	0.007205267
RPUSD3	1.213982394	0.038305741
MRPS23	1.213129453	0.019923311
CCDC137	1.212362452	0.037526789
LOC146880	1.211967758	0.006982047
MXD3	1.211173535	0.045766351
MTHFD2	1.207933377	0.035268512
WDR76	1.206870716	0.042207715
CYB5D1	0.833062861	0.042896734
SIK3	0.832976989	0.036524081
SPATA6	0.831691943	0.048324368
KLHL5	0.819666279	0.036807217
GPD1L	0.819500782	0.036416349
TNK1	0.818761621	0.027056068
IFT46	0.815780586	0.011039893
ZNF167	0.815031021	0.027617227
STARD13	0.81472732	0.029511339
SLC30A1	0.812386589	0.045660908
IQCK	0.812166378	0.014383982
DIXDC1	0.81077698	0.010416232
ZNF577	0.810406005	0.038809852
ZNF717	0.805027533	0.012751427

CISH	0.804502381	0.040912442
EIF4E3	0.801324493	0.001055254
C17orf97	0.79920587	0.038305741
ZNF470	0.795464064	0.001358453
ZFP28	0.795398786	0.001055254
FDXACB1	0.794177769	0.00301687
MAP3K1	0.792460642	0.04773522
FRY	0.79023967	0.042774728
BTRC	0.7888527	0.019564492
RORC	0.786756555	0.038305741
ZNF626	0.786587851	0.026454098
ZNF583	0.784811868	2.49097E-05
C14orf45	0.782010607	0.020733852
ZSCAN18	0.778632932	0.006582365
ZNF502	0.777098168	0.027617227
TTC12	0.76637057	0.004973296
ARMCX1	0.763416962	0.045289603
SGK223	0.759551314	0.043385438
VWA5A	0.755728103	0.04773522
SIAE	0.755328114	0.007727415
HPN	0.754026976	0.037570163
TMEM25	0.746392445	0.004542686
ZNF285	0.743610792	0.006796562
IQGAP2	0.732529684	0.040558706
PDE6B	0.731747593	0.014318591
COL4A5	0.727230898	0.040314929
UBXN10	0.722253484	0.030172337
KIF13B	0.722168188	0.011039893
ABCA3	0.721141487	0.011690184
ZNF471	0.718712678	0.007205267
MUC1	0.717467985	0.041784994
ZNF677	0.708672844	0.011690184
NOSTRIN	0.706731126	0.019564492
DYNC2H1	0.706406259	0.004583512
C4A	0.704987882	0.042321625
ZNF542	0.698561344	8.28867E-11
CACNA1D	0.698244097	0.024271991
RAI2	0.679138095	0.011936026
WNT5A	0.671563112	0.016068152
ABAT	0.666558726	0.012193158
PTPRT	0.624565861	0.045766351
ZNF582	0.607994039	1.02695E-19
ZNF238	0.607342697	0.009851137

NXNL2	0.599486144	0.027653933
IGFALS	0.443953084	0.011690184

Supplementary Table S4. Correlations between *ZNF582-AS1* expression and DNA methylation in the *ZNF582-AS1* promoter 13 CpG sites

CpG site	Spearman's correlation	P value
cg01772700	-0.197	**1.59E-05**
cg24733179	-0.339	**3.9E-14**
cg11740878	-0.257	**1.5E-08**
cg09568464	-0.443	**4.55E-24**
cg02763101	-0.384	**4.8E-18**
cg22647407	-0.333	**1.05E-13**
cg08464824	-0.361	**5.91E-16**
cg13916740	-0.351	**3.63E-15**
cg24039631	-0.304	**1.51E-11**
cg20984085	-0.38	**1.28E-17**
cg25267765	-0.437	**2.05E-23**
cg07135042	-0.453	**3.17E-25**
cg07778983	-0.091	0.047715

Supplementary Table S5. Predicted miRNA targets on the *ZNF582-AS1* transcripts

miRNA name	Binding site types in different *ZNF582-AS1* transcripts	Positions in *ZNF582-AS1* transcripts	Fold change in expression	Adjusted p value
hsa-miR-130b-5p	ENST00000585659 (variant 2)		0.774	0.014
	7mer	493-510		
	6mer	1191-1215		
	6mer	282-299		
hsa-miR-590-5p	ENST00000587979 (variant 1)		0.730	5.16E-05
	8mer	1233-1242		
	7mer	1294-1303		
	ENST00000585659 (variant 2)			
	8mer	1222-1231		
	7mer	1283-1292		
hsa-miR-627-3p	ENST00000587979 (variant 1)		0.825	0.012
	8mer	908-928		
	8mer	764-781		
	6mer	1170-1192		
	ENST00000585659 (variant 2)			
	8mer	491-516		
	8mer	897-917		
	8mer	753-770		
	6mer	1159-1181		
	7mer	536-547		
hsa-miR-939-3p	ENST00000587979 (variant 1)		0.735	1.26E-04
	7mer	1460-1481		
	7mer	551-573		
	6mer	1308-1331		
	6mer	801-821		
	ENST00000585659 (variant 2)			
	7mer	1449-1470		
	7mer	312-334		
	6mer	1297-1320		
	6mer	790-810		
	ENST00000589888 (variant 3)			
	7mer	1128-1149		
	7mer	729-751		
	6mer	979-999		
hsa-miR-940	ENST00000587979 (variant 1)		0.719	0.011
	7mer	1594-1600		
	7mer	1435-1460		
	6mer	973-999		

	6mer	1545-1551		
	ENST00000585659 (variant 2)			
	7mer	1583-1589		
	7mer	1424-1449		
	6mer	962-988		
	6mer	1534-1540		
	ENST00000589888 (variant 3)			
	7mer	1262-1268		
	7mer	1103-1128		
	6mer	1213-1219		
hsa-miR-3682-3p	ENST00000587979 (variant 1)		0.766	0.005
	8mer	1374-1390		
	8mer	1399-1414		
	ENST00000585659 (variant 2)			
	8mer	1363-1379		
	8mer	1388-1403		
	ENST00000589888 (variant 3)			
	8mer	1042-1058		
	8mer	1067-1082		
hsa-miR-4746-5p	ENST00000587979 (variant 1)		0.681	5.42E-05
	8mer	166-185		
	6mer	716-734		
	ENST00000585659 (variant 2)			
	8mer	152-171		
	6mer	699-723		
	ENST00000589888 (variant 3)			
	8mer	181-200		
	6mer	894-912		

Supplementary Table S6. Predicted transcription factors for *ZNF582-AS1*

Transcription Factors	TSS position of ZNF582-AS1	Binding Site Start	Binding Site End	Cell lines	Reference description
CTCF	chr19:56393655	chr19:56393109	chr19:56393646	MCF7	Schmidt D, et al. Genome Res 2010
KDM5B	chr19:56393655	chr19:56393448	chr19:56393572	T-47D	Yamamoto S, et al. Cancer Cell 2014
	chr19:56393655	chr19:56393254	chr19:56393354	T-47D	Yamamoto S, et al. Cancer Cell 2014
HIF1A	chr19:56393655	chr19:56393150	chr19:56393909	T-47D	Zhang J, et al. EMBO J 2015
ARNT	chr19:56393655	chr19:56393174	chr19:56393888	T-47D	Zhang J, et al. EMBO J 2015
NRF1	chr19:56393655	chr19:56393152	chr19:56393920	T-47D	Zhang J, et al. EMBO J 2015
